# Involvement of the Gut Chemosensory System in the Regulation of Colonic Anion Secretion

**DOI:** 10.1155/2015/403919

**Published:** 2015-03-19

**Authors:** A. Kuwahara

**Affiliations:** Laboratory of Physiology, School of Food and Nutritional Sciences/Graduate Division of Nutritional and Environmental Sciences, University of Shizuoka, Shizuoka, 422-8526, Japan

## Abstract

The primary function of the gastrointestinal (GI) tract is the extraction of nutrients from the diet. Therefore, the GI tract must possess an efficient surveillance system that continuously monitors the luminal content for beneficial or harmful compounds. Recent studies have shown that specialized cells in the intestinal lining can sense changes in this content. These changes directly influence fundamental GI processes such as secretion, motility, and local blood flow via hormonal and/or neuronal pathways. Until recently, most studies examining the control of ion transport in the colon have focused on neural and hormonal regulation. However, study of the regulation of gut function by the gut chemosensory system has become increasingly important, as failure of this system causes dysfunctions in host homeostasis, as well as functional GI disorders. Furthermore, regulation of ion transport in the colon is critical for host defense and for electrolytes balance. This review discusses the role of the gut chemosensory system in epithelial transport, with a particular emphasis on the colon.

## 1. Introduction

The primary function of the gastrointestinal (GI) tract is obtaining energy sources from the diet as nutrients. Under physiological conditions, approximately 8 L of fluid is secreted into the small intestine per day. However, the majority of the secreted fluid (~6-7 L/day in humans) is reabsorbed in the small intestine, with approximately 1.5 L of chyme normally passed through the ileocecal valve into the large intestine daily [1]. Less than 100 mL of fluid is excreted outside the body with the feces. The main functions of the colon are the salvage of the remaining fluid and electrolytes entering from the small intestine and the dehydration and storage of feces. However, colonic epithelia are also able to secrete fluid as a host defense mechanism.

In the colon, approximately 100 trillion bacteria, termed the gut microbiota, are present in the lumen. Gut microbiota continuously produces large quantities of bioactive chemicals that can be beneficial or harmful to the host, as confirmed by analysis of the microbiota genome. Such analysis estimated that gut microbiota contains 150-fold more genes than does the host genome [2]. These bioactive chemicals have a profound influence on many aspects of human health, as gut microbiota are able to produce harmful substances, in addition to those that are beneficial. For example, bacterial fermentation of indigestible carbohydrates produces short-chain fatty acids (SCFAs), which can affect various GI functions including ion transport and motility. These SCFAs can be absorbed by the colonic mucosa as energy sources [3]. On the other hand, bile acids entering the colon can be metabolized by microbiota; thus, primary bile acids are converted to secondary bile acids [4]. Secondary bile acids can subsequently promote the development of GI malignancies [5]. Therefore, the colon must be able to discriminate between beneficial and deleterious substances.

One of the more important host-defense mechanisms of the colon is the ability to flush out harmful substances via fluid secretion, mainly through Cl^−^ secretion. Indeed, the colon is able to adapt to extreme changes in electrolyte flow such as those during severe secretory or reabsorptive states [1, 6]. Under physiological conditions, there is net colonic absorption of Na^+^ and Cl^−^ in the colon. The mechanism underlying colonic Cl^−^ secretion is well understood and includes the following components: the luminal cystic fibrosis transmembrane conductance regulator (CFTR), which is a Cl^−^ channel that is activated by cAMP; basolateral Cl^−^ uptake via a Na^+^-K^+^-2Cl^−^ cotransporter (NKCC1); and Ca^2+^-activated basolateral K^+^ channels that recycle K^+^ and provide the driving force for Cl^−^ secretion. The secretion of Cl^−^ into the colonic lumen, followed by a paracellular flux of Na^+^, induces fluid secretion via an osmotic gradient. The energy for active Cl^−^ transport is provided by the action of Na^+^-K^+^ ATPase. Physiological or pathophysiological stimuli of Cl^−^ secretion act on one or more of these components. In essence, switching between absorption and secretion is controlled by the enteric nervous system (ENS) and by a large number of hormones that usually bind to their respective receptors on the basolateral membrane. In addition to these control systems, recent studies have shown that luminal bioactive substances produced by gut microbiota, including SCFAs, affect epithelial ion transport through the gut chemosensory system [7–9].

The presence of a gut chemosensory system is evident. The same taste transduction molecules that are found in the taste buds of lingual papillae, such as *α*-gustducin, are present in the human and rodent intestinal mucosa [10, 11]. Accordingly, mRNA expression of taste receptor type 1 (T1R) and type 2 (T2R) families in human and rodent GI tracts has been reported [12]. Therefore, the gut chemosensory system has important roles in controlling GI functions including ion transport. However, the contribution of the gut chemosensory system to the regulation of colonic ion transport is not well understood.

This review aims to provide an overview of the involvement of the gut chemosensory system in colonic ion transport and its mechanisms.

## 2. SCFA Receptors

SCFAs are the predominant anions in the content of the large intestine, existing at concentration of ~100 mM and mainly consisting of acetate, propionate, and butyrate. They are produced by bacterial fermentation of specific indigestible dietary fibers and oligosaccharides that are not absorbed in the upper GI tract. SCFAs produced in the large intestine are known to affect a variety of physiological and pathophysiological functions; luminal SCFAs are not only absorbed as nutrients across the intestinal epithelia, but also utilized as chemical signals that influence epithelial proliferation [13], mesenteric blood flow [14], colonic motility [15], and colonic ion transport [16, 17]. For example, luminal application of propionate and butyrate, but not acetate, in the colon has been reported to induce epithelial Cl^−^/HCO_3_^−^ secretion via both neural and nonneural mechanisms [16, 17]. On the other hand, serosal application of propionate and other SCFAs did not elicit Cl^−^ secretion [17]. These results suggest that luminal SCFAs are detected by certain chemosensory systems located in the colonic epithelial layer. Until recently, the mechanism by which luminal SCFAs are detected in the intestinal mucosa and the implications of the SCFA-induced secretory response were unclear.

In 2003, two orphan G-protein coupled receptors, FFA2 (GPR43) and FFA3 (GPR41), were discovered to be receptors for SCFA [18–20]. These two receptors share ~40% amino acid sequence similarity and remain conserved across several mammalian species. They differ in their affinity for SCFAs, their tissue distribution, and their physiological roles. FFA2 has similar affinities for acetate, propionate, and butyrate, whereas FFA3 differs in affinities according to the sequence propionate > butyrate ≫ acetate. Thus, acetate preferentially activates FFA2, propionate primarily activates FFA3, and butyrate activates both FFA2 and FFA3 equally.

FFR2 and FFR3 have distinct G-protein-couples in their intracellular signaling cascades, FFA2 couples to both pertussis toxin-sensitive (G_*i*/*o*_) and -insensitive (G_*q*_) G protein whereas FFA3 only couples to G_*i*/*o*_ protein. We have recently demonstrated that colonic epithelia, particularly, peptide YY and glucagon-like peptide 1- (GLP-1-) containing L-type enteroendocrine cells in humans [21], guinea-pigs [16], and rats [22], express two SCFA receptors, FFA2 and FFA3. Our morphological data suggest that SCFA receptors located on colonic epithelial cells can detect luminal SCFA, thus eliciting secretory responses through neural and nonneural mechanisms.

Segmental heterogeneity of electrolyte transport in the colon has also been previously observed in humans and other species [16, 17, 23]. In the case of SCFAs, propionate and butyrate, but not acetate, induce Cl^−^/HCO_3_^−^ secretion in the rectum, as well as in the distal and middle colon. On the other hand, propionate and butyrate do not stimulate Cl^−^/HCO_3_^−^ secretion in the proximal colon. This regional difference in the secretory responses to luminal propionate can be explained by the regional difference in the acetylcholine (ACh) content and its release in the proximal and distal colon [24].

Luminal application of propionate in the distal colon induces Cl^−^/HCO_3_^−^ secretion, and pretreatment of the mucosal surface with procaine or superficial mucosal damage with hypertonic sodium sulfate or xylose inhibits the propionate-induced secretion by 90% [17, 24]. Therefore, propionate-induced Cl^−^/HCO_3_^−^ secretion is caused by the activation of SCFA receptors located on mucosal epithelial cells.

Neural blockade with tetrodotoxin (TTX) inhibits the propionate-induced Cl^−^/HCO_3_^−^ secretion by 40% compared with the control, whereas atropine and local anesthesia remarkably reduce propionate-induced responses by 81–90% and 76–82%, respectively [17, 24]. Furthermore, propionate-induced Cl^−^/HCO_3_^−^ secretion is not affected by tachyphylaxis, calcitonin gene-related peptide, 5-hydroxytryptamine (5-HT), histamine, neurotensin, or substance P [25]. The GI tract is densely innervated by cholinergic neurons, and Cl^−^/HCO_3_^−^ secretion is induced by activation of muscarinic receptors located on colonic epithelial cells [26, 27]. These observations suggest that SCFA-induced Cl^−^/HCO_3_^−^ secretion is linked to ENS, with involvement of cholinergic secretomotor neurons and nonneural release of ACh.

**Figure 1:**
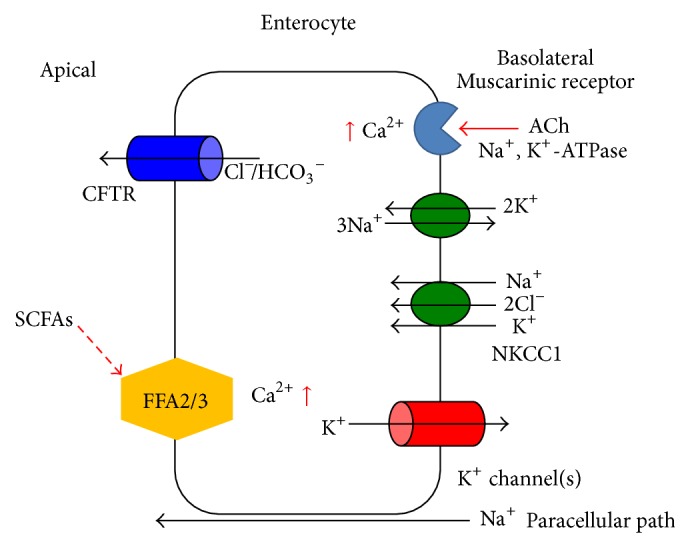
Schematic diagram of Cl^−^ secretion stimulated by short-chain fatty acids in colonic epithelial cells. At the basolateral membrane, Cl^−^ enters the cell from the basolateral space across the Na^+^, K^+^-2Cl^−^ cotransporter (NKCC1). Na^+^, K^+^-ATPase causes drainage of Na^+^, and K^+^ leaves via the K^+^ channel. Na^+^ also moves to the apical side paracellularly. The cystic fibrosis transmembrane conductance regulator (CFTR), which is located on the apical membrane, allows Cl^−^ to exit the cell. The colonic epithelium CFTR Cl^−^ conductance is constitutively active [1]. Luminal SCFAs stimulate the FFA2/3 receptors located on the apical membrane, which in turn activate second messenger pathways to induce ACh release from epithelial cells basolaterally. The released ACh activates muscarinic receptors located on the basolateral membrane of epithelial cells, inducing Cl^−^ secretion.

Recently, Yajima et al. showed that ACh is released from the basolateral side of the distal colon by luminal chemical stimulation with SCFA concomitant with propionate-induced Cl^−^/HCO_3_^−^ secretion [24]. Therefore, the remaining 50% of propionate-induced Cl^−^/HCO_3_^−^ secretion may be due to the release of ACh from the epithelial cells into the basolateral side. In the same study, Yajima et al., showed that prior addition of luminal 3-Cl^−^ propionate completely blocked the short-circuit current (*I*_sc_) response and abolished ACh release in response to luminal propionate. They concluded from the results that ACh-storing epithelial cells have a receptor for propionate although further studies are necessary to identify specific cell that store ACh. Therefore, ACh release stimulated by FFAs may affect Cl^−^/HCO_3_^−^ secretion by autocrine fashion (Figure 1).

With respect to the involvement of SCFA receptors, FFA3 may be involved in the secretory process since acetate, the preferred ligand of FFA2, has no effect on mucosal Cl^−^/HCO_3_^−^ secretion in distal colon of rats [17]. Unfortunately, the intracellular molecular pathways underlying the effects of SCFAs on colonic Cl^−^/HCO_3_^−^ secretion are still not fully understood. Therefore, further study is needed to identify the molecular pathways of FFA-stimulated ion transport in the colon.

Indigestible dietary fibers are fermented in the cecum and in the proximal colon by anaerobic microbiota, as mentioned previously. Most bacterial activity occurs in the cecum and in the proximal colon, where substrate availability is highest, with the availability of substrates declining toward the distal colon [28]. Therefore, the proximal colon is continuously exposed to high concentrations of SCFAs, which decrease from the proximal colon to distal colon. However, the proximal colon does not secrete Cl^−^/HCO_3_^−^ in response to SCFAs, as mentioned above. On the other hand, the distal colonic mucosa is exposed to SCFAs when semisolid contents containing SCFAs are transported to the distal colon. Therefore, detection of SCFAs is important in the distal colon as it has ability to secrete Cl^−^/HCO_3_^−^ after SCFA stimulation.

In combination with the contractile response, the secretory response to luminal SCFAs in the distal colon seems to function as a lubricant for the movement of luminal contents in the colon. Furthermore, the distal colon and rectum are a boundary between the host and external environment; thus, the high secretory ability of the distal colon is physiologically important for host defense, as it needs to flush out harmful agents, in addition to finalizing electrolyte tuning.

## 3. Bitter Taste Receptors

In recent years, numerous studies have suggested the presence of taste receptors and taste-associated signaling components in the GI tract, in addition to their presence in the gustatory system [29]. The discovery of taste-associated molecules in the GI tract has led to the hypothesis that taste receptors are a part of the gut chemosensory system that recognizes nutrients and chemicals, which enter the GI tract (e.g., FFA2 and FFA3), and trigger various physiological processes [30]. The bitter taste, one of the five basic tastes, is mediated by bitter taste receptors (T2Rs). The bitter taste signal is a “notifier” of toxic substances, allowing the host to avoid harm [31]. Genomic sequencing analysis has identified the T2R family as a receptor family specific to bitter tastants, consisting of ~30 members in humans and rodents [32–35].

The *α*-subunit of the taste-specific G protein gustducin is expressed in the GI mucosa of humans and rodents [10, 11, 36, 37]. Unlike FFA2- and FFA3-expressing cells, cells expressing the bitter receptor have not been identified. Several studies in model cell lines [38, 39] and a histochemical study using an antibody raised against mouse T2R138 suggested that enteroendocrine cells express putative bitter taste receptor [40]. However, another study has recently shown that a subset of mouse colonic goblet cells also express the bitter taste receptor T2R131 [41]. Since goblet cells produce mucus to protect intestinal epithelia [42, 43], T2R-expressing cells may contribute defense-related functions involving the recognition of harmful bioactive chemicals.

Recently, we have shown that, in mucosa-submucosa preparations mounted in Ussing chambers, the mucosal application of 6-*n*-propyl-2-thiouracil (6-PTU) at concentrations greater than 10^−4^ M increased *I*_sc_ in both human and rat colons in a concentration-dependent manner [7]. Multiple T2R family members (at least T2R-1, -4, and -38) in humans are known to detect 6-PTU [34, 44, 45], and these genes are the most conserved between humans and rodents [12]. Previous human taste-test studies and brief-access mouse studies have also shown that the minimal effective concentration of 6-PTU is ~10^−4^ M [31, 46, 47]. Therefore, the threshold for 6-PTU in T2R-expressing cells in the colon in both humans and rats is similar to that in gustatory senses. After the addition of 6-PTU, the base line *I*_sc_ gradually increased and reached a plateau over 10–15 min, which continued for >20 min [7]. The increase in *I*_sc_ induced by 6-PTU was reduced by bumetanide (10^−4^ M), an inhibitor of NKCC1, to 69% of the control, whereas NPPB (10^−4^ M), an inhibitor of the CFTR, almost completely abolished the 6-PTU-induced increase in *I*_sc_. NPPB-sensitive Cl^−^ channel, for example, CFTRs located at the apical membrane, also secretes HCO_3_^−^ [1]. Thus, the 6-PTU-induced increase in *I*_sc_ is due to secretion of Cl^−^ and HCO_3_^−^. This is further supported by the observation that the 6-PTU-induced *I*_sc_ response is almost completely abolished in Cl^−^/HCO_3_^−^-free solution [7].

The 6-PTU response elicited is reduced by piroxicam, a nonselective COX inhibitor, and NS-398, a COX-2 inhibitor, but is not affected by TTX. Therefore, 6-PTU- stimulated anion secretion is thought to be involved in prostaglandin (PG) synthesis [7]. Furthermore, exogenous addition of prostaglandin E_2_ (PGE_2_) enhances 6-PTU-induced *I*_sc_ response in the presence of piroxicam in a concentration-dependent manner [7], indicating that the 6-PTU-induced increase in *I*_sc_ may be amplified when the concentration of extracellular PGE_2_ in the colon is elevated, for example, during inflammation. The PGE_2_ concentration in the intestine can be increased by mechanical stimulation [48] or inflammation [49, 50], with PGE_2_ concentrations over 10^−7^ M considered pathophysiological [51]. Therefore, the 6-PTU-induced fluid secretion in the presence of high PGE_2_ concentrations is considered to be a host defense mechanism to flush out noxious substances from the colonic lumen, during, for instance, inflammation.

**Figure 2:**
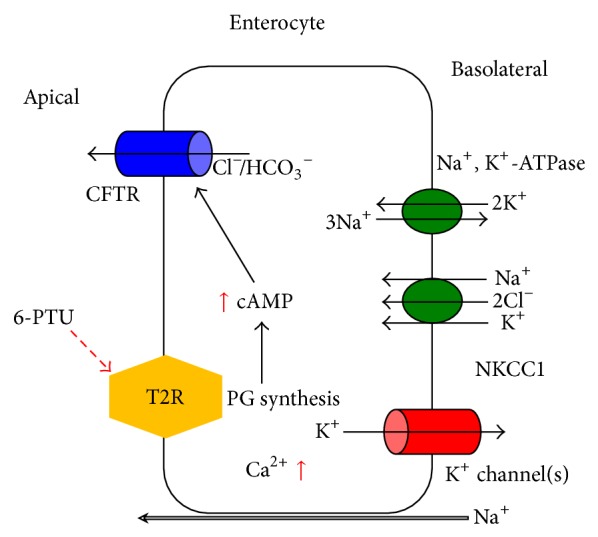
Schematic diagram of Cl^−^ secretion stimulated by a bitter tastant (6-PTU) in colonic epithelial cells. Activation of apical T2R by luminal bitter tastant results in the synthesis of prostaglandin. This prostaglandin then induces an increase in intracellular cAMP concentration ([cAMP]_*i*_). Elevated [cAMP]_*i*_ activates the CFTR Cl^−^ channels to mediate Cl^−^/HCO_3_^−^ secretion. Activation of T2R simultaneously causes an increase in [Ca^2+^]_*i*_. The elevated [Ca^2+^]_*i*_ modulates the Ca^2+^-activated basolateral K^+^ channels, providing a driving force for the exit of Cl^−^/HCO_3_^−^.

PGE_2_ is known to increase the concentration of intracellular cAMP in colonic epithelial cells [52]. It has also been reported that STC-1, a mouse enteroendocrine cell line, expresses T2R mRNA and that 10^−3^ M 6-PTU increases the intracellular Ca^2+^ concentration ([Ca^2+^]_*i*_) [12]. These results raise the possibility that bitter tastants (including 6-PTU) that induce an increase in [Ca^2+^]_*i*_ in colonic epithelia and that elicit Cl^−^/HCO_3_^−^ secretion do so via interactions with PGs (Figure 2).

The mRNA expression of human T2R-1, T2R-4, and T2R-38, as well as orthologous rat T2R-1, T2R-16, and T2R-26, is detected in the colonic mucosa by real-time PCR (RT-PCR) [7]. Although which specific receptor responds to 6-PTU has not been determined, many members of the T2R family that can detect 6-PTU are expressed in the colon [11]. These results suggest that 6-PTU may be detected by colonic epithelial T2R in both humans and rats, although the precise cellular localization of T2R is currently unknown.

With respect to its physiological significance, bitter tastant-induced anion secretion in the colon is considered an important mechanism to flush out noxious agents from the colonic lumen. For example, bitter compounds that enter the large intestine under normal conditions are most frequently bile acids and their bacterial metabolites. As secondary bile acids promote tumors [53], bitter sensing in the large intestine may be a necessary mechanism for host defense.

## 4. Odorant Receptor (OR)

The colonic mucosa of both humans and rats express OR mRNA, and luminal odorants induce 5-HT secretion in isolated duodenal enterochromaffin (EC) cells and in EC cell lines [54, 55]. Thymol, a major odor constituent of edible herbs that is used in oral care products, activates certain types of the apical odorant receptor (OR1G1). Specifically, it activates class II (the terrestrial-type OR group) but not class I (the fish-like OR group) OR1G1 [56]. Since bacteria have the capacity to synthesize isoprene units and terpenoid biosynthesis enzymes [57, 58], active odor molecules may be produced in the mammalian colon. Indeed, a great variety of volatile compounds (including acids, alcohols, aldehydes, and terpenoids) are detected in human feces [59]. It has been reported that the concentration range of fecal indole is 0.5–1 mM in healthy men [60, 61]. Therefore, the monitoring of volatile compounds in the colonic lumen is critical for host defense.

**Figure 3:**
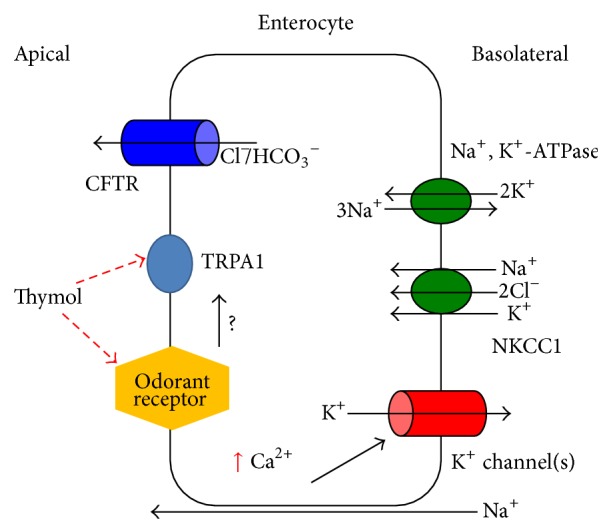
Schematic diagram of Cl^−^ secretion stimulated by an odor tastant (thymol) in colonic epithelial cells. Activation of OR1G1 by luminal thymol increases [Ca^2+^]_*i*_. The elevated [Ca^2+^]_*i*_ may modulate Ca^2+^-activated basolateral K^+^ channels, providing a driving force for the exit of Cl^−^/HCO_3_^−^. Activation of OR1G1 may also activate TRPA1 although it is still unclear whether ORs are linked to TRPA1.

Recently, we showed that mucosal addition of thymol (10^−3^ M) induces Cl^−^ and HCO_3_^−^ secretion in a concentration-dependent manner in both the human and rat colon [8]. Addition of TTX (10^−6^ M) or piroxicam (10^−5^ M) did not affect this response, suggesting that thymol-induced anion secretion is independent of the neural and PG synthesis pathways. This differs from stimulation of the bitter taste receptor; thus, there are distinct mechanisms for detecting tastants in the colonic mucosa. It has been reported that odorant stimulation leads to an increase in [Ca^2+^]_*i*_ in olfactory neurons and in other OR-expressing cells, depending on extracellular Ca^2+^ [54, 62, 63]. Thymol-induced electrogenic anion secretion is also abolished under Ca^2+^-free conditions [8]. These results suggest that extracellular Ca^2+^ is required to elicit thymol-induced anion secretion in the large intestine (Figure 3).

Thymol-induced anion secretion in the distal colon is reduced by HC-030031, a transient receptor potential A1 (TRPA1) blocker. Furthermore, TRPA1 mRNA is detected in the isolated mucosa of humans and rats [8, 64]. Several odor molecules, particularly those present in spices, are known ligands of not only GPCRs, but also the transient receptor potential (TRP) channel. Thymol activates transient receptor potential vanilloid 3 (TRPV3) and TRPA1 in a cell-expression system [65, 66]. In the GI tract, it has been reported that TRPA1 activity is involved in the motility of the small intestine [67]. Together, these results suggest that thymol-induced electrogenic anion secretion is mediated via the TRPA1 channel. In addition, thymol has been reported to affect luminal SCFA-induced ion secretion. Propionate-induced increases in *I*_sc_ are almost completely abolished by pretreatment of tissues with thymol [8]. Therefore, the physiological effects of luminal SCFAs in the large intestine are likely to be modulated by luminal odorant chemicals.

Pretreatment of tissues with bumetanide or Cl^−^ free solution attenuated the thymol-induced increase in *I*_sc_. Consistent with other studies, the absence of HCO_3_^−^ and Cl^−^ completely suppressed the *I*_sc_ response to thymol. Together, these results indicate that the thymol-induced increase in *I*_sc_ involves electrogenic Cl^−^ and HCO_3_^−^ secretion in a NKCC1-dependent manner.

It has been reported that thymol-induced electrogenic anion secretion is mediated by the cholinergic neural pathway in the porcine small intestine [68]. However, the mechanisms of thymol-induced secretion are likely different in the small and large intestine as thymol-induced anion secretion is not blocked by TTX in the rat or human large intestine [8]. This discrepancy may be attributed to the following reasons. First, thymol-induced anion secretion in the small intestine is involved in the release of 5-HT because duodenal EC cells release 5-HT after OR stimulation [54, 55]. Second, thymol-induced anion secretion is not blocked by 5-HT_3_ and 5-HT_4_ receptor antagonists in the large intestine [8]. Therefore, luminal thymol-induced anion secretion in the large intestine is mediated by nonneural and nonserotonergic pathways in rats and in humans.

As bacteria can synthesize isoprene units [57], production of active odor molecules similar to thymol may be possible in the mammalian colon. Thus, colonic mucosa may be exposed to high concentrations of various volatile odorants. Because irritant odors, similar to bitter tastants, are danger signals for animals, ORs can play an important role in the luminal surface of the colon in host defense. Although a RT-PCR experiment showed that OR1G1 and TRPA1 are present in both human and rat colonic mucosa [8], colonic epithelia consist of many different cell types, including absorptive, goblet, enteroendocrine, and caveolated cells. Therefore, studies should be done to identify the specific sensor cells expressing ORs and TRPA1. At present, it is still unclear whether OR1G1 is directly involved in thymol-induced anion secretion and whether ORs are linked to TRPA1.

## 5. TRP Channels

The TRP channel member, TRPA1 (also known as ANKTM1), was first identified as a cold-sensitive cation channel in murine sensory neurons and is thought to have a role in nociception [69]. Since multiple environmental irritants can activate TRPA1, TRPA1 functions as a chemosensor in nociceptive neurons [70], in the rat urinary bladder [71], and in human keratinocytes [72]. Endogenous inflammatory mediators can also activate TRPA1 [73–75].

To date, 28 mammalian TRP channels have been cloned and characterized. They are grouped into six subfamilies on the basis of their amino acid sequence homology, namely, TRP ankyrin (TRPA), TRP canonical (TRPC), TRP melastatin (TRPM), TRP mucolipin (TRPML), TRP polycystin (TRPP), and TRP vanilloid (TRPV). TRPA1 expression in the colon has been demonstrated in humans, mice, rats, and dogs by northern blot analysis and by RT-PCR [64, 76–78]. As described in the section Odorant Receptor, luminal thymol-induced anion secretion involves TRPA1. The function of TRPA1 in the transepithelial ion transport system was examined using a potent TRPA1 agonist allyl isothiocyanate (AITC) [9].

**Figure 4:**
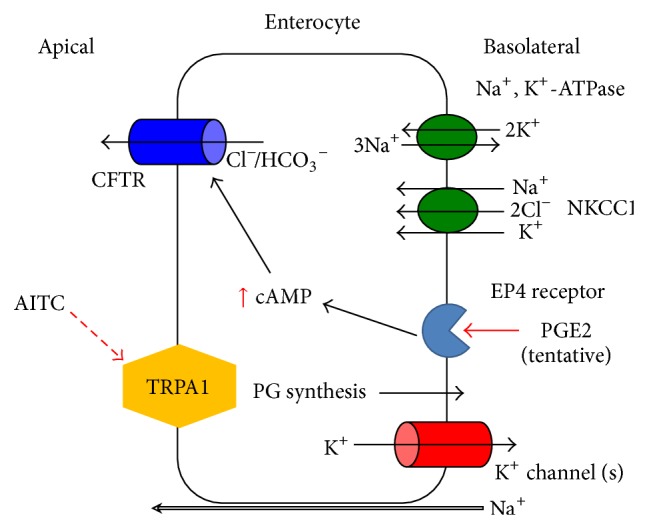
Schematic diagram of Cl^−^ secretion stimulated by TRPA1 in colonic epithelial cells. Activation of apical TRPA1 by luminal AITC causes prostaglandins synthesis. The synthesized PGE_2_ may then be released basolaterally. This released PGE_2_ may act on EP_4_ receptors. Activation of the EP_4_ receptor induces an increase in [cAMP]_*i*_ which then activates the CFTR Cl^−^ channels to induce Cl^−^/HCO_3_^−^ secretion.

In the human and rat distal colon, the addition of AITC (10^−6^–10^−3^ M) to the luminal side induced an increase in *I*_sc_ [9]. On the other hand, serosal application of AITC did not elicit an increase in *I*_sc_. AITC-induced increases in *I*_sc_ are significantly decreased in the absence of Cl^−^ and are abolished in the absence of both Cl^−^ and HCO_3_^−^. Further, NPPB (10^−4^ M) and bumetanide (10^−4^ M) significantly reduced AITC-induced *I*_sc_ increases. These results indicate that transepithelial anion secretion induced by the activation of TRPA1 is dependent on Cl^−^ uptake by NKCC1 and on excretion of Cl^−^/HCO_3_^−^ by Cl^−^ channels at the apical membrane (Figure 4).

In fractions enriched with isolated EC cells from the rat small intestine, 5-HT was released by AITC and cinnamaldehyde [67]. However, in the colon, a 5-HT_3_ antagonist, 3-tropanyl-3, 5-dichlorobenzoate, and a 5-HT_4_ antagonist SB204070 (10^−5^ M) did not affect the response to AITC [9]. Furthermore, coapplication of these antagonists abolished the response to the serosal addition of 5-HT (10^−5^ M) but did not affect the AITC-induced *I*_sc_ response. Serosal application of the TRPA1 blocker HC030031 (10^−4^ M) did not affect the response to AITC, whereas mucosal treatment significantly inhibited the AITC-induced increases in *I*_sc_. These observations support the hypothesis that TRPA1 likely functions on the apical side of colonic epithelia.

COX products are involved in the colonic ion transport system. In particular, PGE_2_ induces Cl^−^ secretion in the rat colon [79] and enhances the effects of other secretagogues [7]. Mucosal treatment with a COX inhibitor, piroxicam (10^−5^ M), decreases the AITC-induced response in the rat and human colon. However, exogenous PGE_2_ does not enhance AITC-induced anion secretion, even in the absence of the COX inhibitor, suggesting that AITC stimulates PGE_2_ synthesis [9].

Although the mechanisms by which PGE_2_ contributes to 6-PTU- and AITC-induced anion secretion differ, PGE_2_ likely plays important roles in the luminal chemosensory system. The function of PGE_2_ in the GI tract has been well studied, especially in relation to its receptors, EP_1_, EP_2_, EP_3_, and EP_4_. Only EP_2_ and EP_4_, which are coupled to the cAMP pathway, mediate PGE_2_-induced colonic secretion, but EP_1_ and EP_3_ do not, as they are coupled to an intracellular Ca^2+^ pathway instead [80]. An EP_4_ selective antagonist (ONO-AE3-208) significantly reduced AITC-induced anion secretion, whereas the EP_1/2_ antagonist AH6809 did not affect the response to AITC, indicating that EP_4_, a PGE_2_ receptor subtype, is involved in AITC-induced anion secretion in both human and rat colons [9]. As application of AITC to the mucosal bathing solution did not result in the release of PGE_2_ into the bathing solution, PGE_2_ produced by epithelial cells might be rapidly used as an autocrine transmitter [9].

Recently, the EP_4_ receptor has been found on the apical membrane of human and rat colonic epithelia via immunohistochemical techniques [81]. Since both serosal and mucosal pretreatment with ONO-AE3-208 abolished the response to AITC, the precise localization of the EP_4_ receptor is still unknown. Overall, the evidence clearly indicates that a TRPA1-PGE_2_-EP_4_ secretory pathway that is independent of the neural reflex exists in both human and rat colonic epithelial cells.

TRPA1-dependent thymol-induced anion secretion requires extracellular Ca^2+^, as described in the section Odorant Receptor. However, the response to AITC was not altered by the removal of extracellular Ca^2+^ [9]. Therefore, AITC-induced anion secretion is mediated by PG synthesis via a Ca^2+^ independent process. These conclusions were further confirmed after observations that AITC does not increase [Ca^2+^]_*i*_ in mouse colonic epithelia [78] and that AITC-induced TRPA1 current does not require extracellular Ca^2+^ in HEK cells [82]. Overall, TRPA1 activation appears to induce PGE_2_ synthesis independently of extracellular Ca^2+^ and can cause anion secretion through the EP_4_ receptor in colonic epithelia.

TRPA1 mRNA is detected in isolated crypts of the rat colon [9]. Furthermore, tyramide-based* in situ hybridization* and immunohistochemistry for TRPA1 demonstrated that TRPA1-expressing cells are localized to the surface epithelium of the rat colon [9]. Recently, it has been reported that activation of TRPA1 inhibits spontaneous contractions and transit by direct activation of myenteric neurons [83]. Therefore, TRPA1 agonist-induced colonic Cl^−^ secretion with inhibition of colonic transient seems to physiologically regulate the movement of luminal content in the colon. In addition, TRPA1 may also play a role in flushing out noxious chemicals via massive fluid secretion.

## 6. Conclusion

As colonic mucosa is continuously exposed to noxious chemicals, including toxic compounds such as bacterial metabolites and the products of oxidative stress, in addition to nutrients, the chemosensory system in the gut is critical for distinguishing the nutrients from the other luminal contents. Therefore, proper fluid secretion in the colon is crucial to flush away noxious chemicals, while maintaining host homeostasis.

Although neural and hormonal involvement in fluid secretion in the colon are well documented, the involvement of the gut chemosensory system in the regulation of colonic ion transport is much less understood. Activation of luminal chemosensory receptors is a primary signal eliciting colonic fluid secretion. Gut luminal chemosensing involving FFA2, FFA3, T2R, OR, and TRPA1 may act as a line of defense against noxious agents, preventing the large intestine from being exposed to these agents. Therefore, the gut chemosensory system is important for maintaining luminal homeostasis. A variety of sensory receptors expressed in the colonic mucosa serve important functions, at least in the anion secretory system, which stimulates fluid secretion. However, the specific mechanisms involved in anion secretion induced by the gut chemosensory system are largely unknown. Therefore, more studies are required to define the involvement of the gut chemosensory system in colonic ion transport.
